# Regulatory mechanisms of the early phase of white adipocyte differentiation: an overview

**DOI:** 10.1007/s00018-022-04169-6

**Published:** 2022-02-20

**Authors:** M. Audano, S. Pedretti, D. Caruso, M. Crestani, E. De Fabiani, N. Mitro

**Affiliations:** DiSFeB, Dipartimento di Scienze Farmacologiche e Biomolecolari, Università degli Studi di Milano, 20133 Milan, Italy

**Keywords:** Adipogenesis, Obesity, Transcriptional control of differentiation, Epigenome modifications, Cytoskeleton

## Abstract

The adipose organ comprises two main fat depots termed white and brown adipose tissues. Adipogenesis is a process leading to newly differentiated adipocytes starting from precursor cells, which requires the contribution of many cellular activities at the genome, transcriptome, proteome, and metabolome levels. The adipogenic program is accomplished through two sequential phases; the first includes events favoring the commitment of adipose tissue stem cells/precursors to preadipocytes, while the second involves mechanisms that allow the achievement of full adipocyte differentiation. While there is a very large literature about the mechanisms involved in terminal adipogenesis, little is known about the first stage of this process. Growing interest in this field is due to the recent identification of adipose tissue precursors, which include a heterogenous cell population within different types of adipose tissue as well as within the same fat depot. In addition, the alteration of the heterogeneity of adipose tissue stem cells and of the mechanisms involved in their commitment have been linked to adipose tissue development defects and hence to the onset/progression of metabolic diseases, such as obesity. For this reason, the characterization of early adipogenic events is crucial to understand the etiology and the evolution of adipogenesis-related pathologies, and to explore the adipose tissue precursors’ potential as future tools for precision medicine.

## Introduction

Adipose tissue is found in virtually all living organism, from *C. elegans* to *H. sapiens*. Adipocytes have unique characteristics that scientists have learned especially in the last 20 years, and we now know that their function is essential from many perspectives, such as physical support for organs, endocrine activity, energy balance, and developmental regulation [1, 2]. For this reason, dysregulated adipocytes play a key role in many diseases such as obesity, cancer, and cardiovascular events [2, 3]. In recent years, great attention has focused on the characterization of the molecular mechanisms involved in the generation of new adipocytes, a process known as adipogenesis, and on how these mechanisms may impact physiological processes, as well as the course of different diseases [4, 5].

The purpose of this review is to recapitulate advances in the field of adipogenesis, describing the main molecular mechanisms involved in the early stage of this process. Furthermore, we will describe the main pathological contexts involving alterations of adipogenesis and the main in vivo and in vitro technical approaches to investigate new players in adipocyte differentiation. Also, we will review new therapeutic approaches that target these alterations, with the aim to counteract the course of several diseases.

## Overview on adipogenesis

Adipose tissues are classified in two main types, namely white adipose tissue (WAT) and brown adipose tissue (BAT). WAT is mostly localized in the abdominal cavity surrounding organs and in the subcutaneous area underneath the skin; BAT is predominantly distributed in the interscapular region. Of note, WAT represents the main adipose tissue in humans as it accounts for 10–15% and 25% of total body weight in normal weight men and women, respectively [6]. Although these tissues share common features such as lipid accumulation and endocrine functions, BAT and WAT show significant differences. BAT exhibits multi-locular and smaller lipid droplets compared to WAT due to the high lipid burning for non-shivering thermogenesis, which needs a continue recycling of fatty acids between mitochondrial β-oxidation and cytosolic biosynthesis [7–9]. On the other hand, WAT represents one of the main body energy reservoir where fatty acids are stored as triglycerides [8].

From a developmental point of view, BAT and WAT show significant differences, as they originate from separate cell lineages. BAT adipocytes, like skeletal muscle myocytes, derive from myogenic factor 5 (Myf5) positive progenitors, and express PR/SET Domain 16 (PRDM16), while WAT cells originate from Myf5 negative stem cells [10, 11]. The differentiation of new adipocytes, especially white adipocytes, has been associated to several physiological processes, such as systemic metabolic homeostasis [12] and tissue regeneration [13–15], but also to pathological conditions like obesity [16], cancer [4, 17], metastasis invasion [18, 19] and cardiovascular diseases [20, 21]. For these reasons, in the last 20 years several investigations analyzed the molecular mechanisms involved in white adipocyte differentiation. From a molecular point of view, the adipogenic process involves a plethora of cellular players and activities, which coordinate the transition of adipose tissue precursors/stem cells to preadipocytes and ultimately to mature adipocytes. The adipogenic program consists of two distinct phases: the commitment phase, under specific physicochemical signals, mesenchymal precursor cells limit their commitment to the adipocyte lineage, turning into preadipocytes without significant morphological changes [22, 23]. This irreversible phase is followed by adipocyte differentiation, in which committed preadipocytes first undergo at least two rounds of cell division, namely the mitotic clonal expansion, followed by morphological changes allowing the accumulation of lipids [24–26].

### Adipose tissue stem cells identification

The identification of adipose tissue stem cells raised great interest owing to the poor understanding of mechanisms controlling adipocyte number in pathophysiology [27]. In recent years, a great effort has been made in the characterization of the adipose tissue stem cell subtypes and of the mechanisms controlling their stemness and cell fate. Negative selection to remove fibroblasts (CD31^+^) and immune cells (CD45^+^) from the stromal vascular fraction (SVF) associated to positive isolation of stem cell antigen positive cells (SCA1^+^) by fluorescence-activated cell sorting have been firstly used to isolate white adipose tissue stem cells [28]. Furthermore, CD24^+^ and CD24^−^ cells helped to discriminate between stem cell-like progenitors and committed preadipocytes, respectively [28]. In mice, most white adipocytes originate from a pool of progenitors that are committed either prenatally or early postnatally, and are found close to blood vessels, strongly suggesting that these cells require high oxygen levels, growth factors and metabolites from bloodstream to properly proliferate and differentiate [29]. CD34 and CD36 were also identified as specific markers of fat progenitors [30, 31]. The abundance of CD34 has been correlated with the turnover capacity of white adipocytes in humans. Indeed, similarly to CD34^low^ cells, CD36^+^ progenitors have been demonstrated to be extremely efficient in triglyceride accumulation, with low lipid turnover and a high adipogenic potential, while CD34^high^ cells show high adipogenic potential associated to great triglyceride turnover [32, 33]. Also, platelet-derived growth factor receptor α and β (Pdgfrα and Pdgfrβ) have been demonstrated to play a role in the commitment of adipocyte progenitors [34]. Specifically, gene expression of these two receptors follows two different temporal profiles during adipocyte differentiation in visceral and subcutaneous WAT (sWAT), where Pdgfrα precedes Pdgfrβ expression. Gao and colleagues also showed that thermoneutrality, a condition in which thermogenesis is inactive, and high-fat diet induce Pdgfrβ to favor white adipocyte development at the expense of Pdgfrα positive beige adipocytes, the brown-like adipocytes present in the white adipose tissue [34]. An elegant lineage tracing study combined with functional analyses further characterized the role of Pdgfrα and Pdgfrβ in determining the fate of adipose progenitors [35]. The authors showed that adipocytes originate from the Pdgfrα cell lineage during both postnatal growth and adulthood. On the contrary, adipocytes are only derived from Pdgfrβ positive cells during postnatal growth. In addition, postnatal deletion of Pdgfrα increased adipogenesis, while adult deletion increased β3-adrenergic-receptor-induced beige adipocyte development. Differently, deletion of Pdgfrβ enhances white, brown, and beige adipogenesis [35]. Together, these data, summarized in Table 1, strongly indicate that spatiotemporal patterns of several proteins are crucial in adipogenesis, especially for the commitment of progenitor cells to different adipocyte lineages.

**Table 1:** Summary of markers for the identification and isolation of adipose tissue stem cells

Marker	Description	References
SCA1/Ly6a	Marker of adipose tissue stem cells	[28]
CD24	Committed preadipocytes have been demonstrated to be CD24^−^	[28]
CD34	CD34^low^: high triglyceride accumulation capacity, with low lipid turnover and a high adipogenic potential. CD34^high^: high adipogenic potential associated to great triglyceride turnover	[30, 32]
CD36	Cells expressing this marker show a similar phenotype to CD34^low^ progenitors	[31, 33]
Pdgfrα	Marker of beige adipocytes induced by cold exposure. Associated to pre- and postnatal adipocyte differentiation	[34, 35]
Pdgfrβ	Marker of white adipocytes. Involved only in postnatal adipocyte development	[34, 35]

### Transcriptional control of adipogenesis

In addition to cell surface markers, transcription factors have been widely investigated in adipogenesis. Among them, CCAAT/enhancer-binding protein alpha, beta, delta (C/EBPα, C/EBPβ, C/EBPδ), peroxisome proliferator- activated receptor gamma (PPARγ), sterol regulatory element-binding factor-1c (SREBF1c) and cAMP-response element-binding protein (CREB) have been extensively studied [36, 37]. C/EBP family members have been demonstrated to orchestrate different phases of adipogenesis; C/EBPβ and C/EBPδ, together with CREB, are involved in the first wave of transcription factors responsive to adipogenic stimuli, while C/EBPα, partner of PPARγ, belongs to the second wave involved in the coordination of last stages of adipocyte differentiation [38], and their role are exhaustively reviewed elsewhere [39, 40]. Noteworthy, two isoforms of PPARγ, namely PPARγ-1 and PPARγ-2, have been identified [41]. Specifically, if PPARγ-1 activity is still poorly understood, PPARγ-2 has been demonstrated to be required for white adipogenesis [42]. This information is of great relevance since it indicates that mRNA splicing events are necessary to determine the fate of adipocyte precursor cells [43, 44]. However, mRNA splicing is only one of many cellular processes that have recently been shown to be critical in the early stages of adipogenesis. In the next sections, we will focus on recent biochemical and biological advances involved in this stage of the adipogenic process to highlight the great importance that these mechanisms hold in the development of adipose tissue and related diseases.

## Novel mechanisms involved in early phases of adipogenesis

In the early phase of adipogenesis, the precursor cells undergo a series of modifications that stimulate the expression of the second phase genes, leading to the final differentiation step with lipid-filled adipocytes. Several factors and processes play a key role in driving the transformation of adipocytes precursors. These include *(i)* cell cycle control, which is essential for the mitotic clonal expansion; *(ii)* the rearrangement of the cytoskeleton; and *(iii)* several factors that affect RNA metabolism. This section will highlight new factors or processes that regulate the first step of adipogenesis and then influence the terminal differentiation.

### Cell cycle regulation and mitotic clonal expansion

After adipogenesis induction, adipocyte precursors undergo several rounds of cell division before permanently exiting the cell cycle, a necessary process known as mitotic clonal expansion. Zhao and colleagues [45] have established a new approach to study the cell cycle progression and the timing of commitment to the mature adipocyte phenotype. First, they showed that terminal differentiation does not occur after a precise number of cell cycle divisions, but the commitment takes place in G1, the cell cycle phase characterized by a dramatic increase of PPARγ expression. Then PPARγ slows the cell cycle by increasing transcription of p21 and FKBP Prolyl Isomerase Like (FKBPL), which favors p21 mRNA stability. In addition, PPARγ induces a competitive mechanism between cyclin D1 and p21 expression that controls the number of cells that remain in proliferation and those that exit the cell cycle and proceed to terminal differentiation. This mechanism ensures both the presence of differentiated cells and the maintenance of adipogenic precursors [45].

Several factors and proteins regulate this process by interacting with C/EBPβ [46]. Very recently, a protein belonging to the non-histone chromosomal high mobility group protein family, the High Mobility Group Box 2 (HMGB2), has been studied in the early phase of adipogenesis. The peak of expression of this protein is at 24 h after differentiation induction, while its expression decreases during the late stage. It was then observed that the HMGB2 knockdown, 72 h before adding adipogenic stimuli, impairs adipogenesis while the downregulation 48 h after the induction of adipogenesis does not affect terminal differentiation [46]. Thus, the mechanism by which this protein plays a role during the mitotic clonal expansion is through the binding to the C/EBPβ promoter, stimulating the transcription of factors essential for proper adipocytes differentiation. Conversely, when HMGB2 is removed, the expression of C/EBPβ is lower, and mitotic clonal expansion does not occur properly, causing the formation of smaller adipocytes thus reducing body size in vivo [46].

Another factor involved in the initiation of adipogenesis is Sprouty RTK Signaling Antagonist 1 (Spry1), a negative regulator of MAPK signaling, whose expression increases in adipose tissue stem cells following weight loss in humans [47]. The expression of this protein increases in confluent cells and decreases during the final stage of adipogenesis; the Spry1 knockdown impairs adipocytes’ differentiation and lipid accumulation. By negatively regulating MAPK activity, the authors observed higher phosphorylation and activation of ERK in cells lacking Spry1 at day 1 after adding adipogenic stimuli. In addition, it was previously demonstrated that the MAPK pathway regulates the expression of C/EBPβ [48]. In this context, Spry1 knockdown increased MAPK activity reducing C/EBPβ expression and therefore the adipogenic program [47].

Recently, it has been discovered that Mediator complex subunit 20 (MED20) is required for proper adipogenesis [49]. MED20 is a component of the mediator complex that interacts with RNA polymerase II and this interaction is necessary for the expression of several genes. MED20 can bind some early transcription factors involved in the initiation of adipogenesis, but the strongest interaction is with C/EBPβ. Thus, the mechanism of action by which MED20 plays its role in adipocyte differentiation is by creating a bridge between RNA Pol II and C/EBPβ to control the transcription of PPARγ. Furthermore, knockdown of MED20 in the early stages of adipogenesis inhibits mature adipocyte formation and lipid accumulation in cells, while mice lacking one allele of MED20 in preadipocytes are protected from diet-induced obesity [49]. Nevertheless, the role of MED20 in the physiology of adipose tissue in vivo still requires further investigation.

Moreover, C/EBPβ undergoes post-translational modifications that affect its ability to bind DNA and stimulate transcription of its target genes. For example, poly(ADP-ribose)polymerase-1 (PARP-1) is capable of modifying other proteins through poly(ADP-ribosyl)ation (PARylation). This protein mediates the site-specific PARylation of C/EBPβ, inhibiting the ability of this protein to bind to DNA and stimulate the expression of different genes involved in adipogenesis [50]. Consistently, PARP-1 depletion leads to increased expression of the main adipogenic markers and lipid accumulation. A physiological decrease of PARP-1 levels in the nucleus in the first two days after differentiation induction was observed and it coincides with a decrease of C/EBPβ PARylation, which thus binds to DNA and plays its role as pro-adipogenic factor. Nuclear PARP-1 levels then increase during terminal differentiation when the transcriptional activity of C/EBPβ is no longer observed [50]. These results have been synthesized in Table 2.

**Table 2:** Summary of proteins involved in cell cycle regulation and mitotic clonal expansion during adipogenesis

Protein	Description	References
PPARγ	Master regulator of adipogenesis. It slows the cell cycle by increasing transcription of p21 and FKBPL, hence favoring p21 mRNA stability. PPARγ ultimately induces a competitive mechanism between cyclin D1 and p21 expression controlling the number of cells that remain in proliferation or proceed to terminal differentiation	[45]
HMGB2	Highly expressed in the first 24 h of adipogenesis, it favors the expression of C/EBPβ, cell cycle exit, and adipocyte differentiation	[46]
Spry1	Its expression level increases in the early phases of adipogenesis and acts as a negative regulator of MAPK signaling to favor cell cycle exit and boost the adipogenic program	[47]
MED20	It directly interacts with the RNA polymerase II and C/EBPβ to favor the transcription of PPARγ	[49]
PARP-1	Involved in the PARylation of C/EBPβ, inhibiting its ability to bind to DNA and hence blocking the adipogenic program. Nuclear PARP-1 is required during terminal differentiation to block the transcriptional activity of C/EBPβ	[50]

### Epigenetic and epitranscriptomic control the early phase of adipogenesis

Recent technological advancements in genomics unveiled new aspects of transcriptional regulation during adipogenesis, including epigenetic events. Indeed, histone modifications, e.g., acetylation, methylation, phosphorylation, ubiquitination and DNA and mRNA methylation, have been found to play key roles in the regulation of the adipogenic program (Table 3) [51, 52]. Several enzymes have been studied in this context, like histone deacetylases (HDACs), which allow the removal of acetyl groups from histones, causing chromatin condensation and decreasing gene expression. HDAC3, a member of class I HDACs, has a role during the early phase of adipogenesis [53]. When HDAC3 is silenced at the beginning of differentiation, an increase in adipocyte marker expression and lipid accumulation are observed, while its depletion at the late stage of differentiation is ineffective. Moreover, silencing HDAC3 at the onset of differentiation causes an increase in the expression of genes involved in the browning process, in fatty acid  β-oxidation, and mitochondrial function [53]. These results were also observed in a mouse model where HDAC3 was deleted specifically in adipose tissue [9].

**Table 3:** Summary of epigenetic and epitranscriptomic factors involved in the early phases of adipocyte differentiation

Protein	Description	References
HDAC3	Involved in the removal of acetyl groups from histones, causing chromatin condensation and decreasing gene expression. Its deletion or downregulation increases browning, metabolic activity and differentiation in adipocytes	[9, 53]
SIRT6	It is a member of the sirtuin family of NAD-dependent enzymes that are implicated in cellular stress resistance, genomic stability, aging and energy homeostasis Its downregulation blocks the formation of mature adipocytes	[54]
KDM5A	Activated by C/EBPβ, its downregulation decreases PPARγ and C/EBPα expression and ultimately lipid accumulation. It is involved in the removal of three methyl groups from lysine 4 on H3 (H3K4me3), blunting the expression of target genes	[55]
DNMT1	This enzyme maintains DNA methylation, ensuring the fidelity of this epigenetic patterns across cell divisions. Its downregulation at the onset of differentiation results in decreased formation of mature adipocytes	[56]
FTO	Identified as the first m6a eraser with a recognized role as a genetic factor for obesity, its deletion reduces adipogenic capacity and lipid accumulation	[59]
ZFP217	Its deletion inhibits adipogenesis and lipid accumulation and influences the cell cycle by decreasing cyclin D1 (CCND1) levels, ultimately arresting the mitotic clonal expansion	[62]

Another histone deacetylase, sirtuin 6 (SIRT6), a member of the sirtuin family of NAD^+^-dependent enzymes, has been shown to play a role during the early stage of the adipogenic process [54]. The knockdown of SIRT6 before adipogenesis induction blocks the formation of mature adipocytes, whereas the silencing at one day after adipogenic stimulation does not affect differentiation. The authors suggested that SIRT6 causes cell death due to cell cycle blockage in S and M phases and may regulate the mitotic clonal expansion through inhibition of p27 degradation. Furthermore, SIRT6 regulates Kinesin Family Member 5C (KIF5C) expression by binding to its promoter and reducing the levels of acetylation of lysine 9 and 56 on histone H3(H3K9ac and H3K56ac). These epigenetic modifications cause a decrease in the KIF5C expression during the early stage of adipogenesis, resulting in the formation of mature adipocytes. KIF5C is a negative regulator of the mitotic clonal expansion that inhibits Casein Kinase 2 (CK2) activity by preventing nuclear translocation of CK2α' subunits during adipogenesis, a crucial process for proper mitotic clonal expansion. Given the negative regulation of SIRT6 on KIF5C, this sirtuin appears to be a factor that stimulates the initiation of the mitotic clonal expansion and is involved in the early phase of adipogenesis [54].

Other epigenetic modifications such as lysine methylation of histones are associated with gene activation or repression. The Lysine Demethylase 5A (KDM5A) protein is a target of C/EBPβ. C/EBPβ can induce KDM5A expression 48 h after the induction of the adipogenic program. The downregulation of KDM5A decreases PPARγ and C/EBPα expression and ultimately lipid accumulation. In addition, KDM5A removes three methyl groups from lysine 4 on H3 (H3K4me3), blunting the expression of key genes. Consistently, KDM5A knockdown cells displayed increased levels of Wingless-Type MMTV Integration Site Family, Member 6 (Wnt6) that, by activating β-catenin pathway, negatively affects adipogenesis [55]. In addition, it was discovered that DNA methylation promotes the early phase of adipogenesis [56]. The DNA Methyltransferase 1 (DNMT1) reaches its highest expression 16 h after differentiation induction and declines within 48 h. Usually, methylation of gene promoters by DNMTs causes gene silencing and, due to the trend of Dnmt1 expression during adipogenesis, it was hypothesized that it acts on adipogenic repressors such as Wnt10a. The inhibition of DNA methylation by treatment with the DNMT inhibitor 5-Aza-2'-deoxycytidine or downregulating DNMT1 at the onset of differentiation results in decreased formation of mature adipocytes and increased Wnt10a expression [56].

It is worth noting that, epigenetic modifications affect not only DNA but also RNA [57]. The transfer of a methyl group on the sixth position of the nitrogen (N) atom of the adenine (A) RNA strand is termed RNA N6-methyladenosine (m6a) modification. This modification can also be reverted, and the FTO protein (fat mass and obesity-associated gene) has been identified as the first m6a eraser with a recognized role as a genetic factor for obesity [58]. 3T3-L1 cells were used to verify the role of FTO during adipogenesis. Silencing of FTO inhibited lipid accumulation and caused a reduction in PPARγ and C/EBPα levels, thus hinting that demethylase activity is required for adipogenesis [59]. To understand how FTO affects the process of adipogenesis, MEFs derived from FTO knock-out (KO) mice were induced to differentiate. The results obtained show a reduced adipogenic capacity due to decreased expression of key adipogenic genes resulting in blunted lipid accumulation. On the contrary, MEFs derived from FTO-overexpressing mice showed an opposite effect on the generation of mature adipocytes, exhibiting increased adipogenic capacity [59]. From the mechanistic point of view, the authors showed that FTO promotes the mitotic clonal expansion by regulating the splicing of RUNX1 Partner Transcriptional Co-Repressor 1 (RUNX1T1, S form), which enhances adipogenesis [60]. A recent study by Wu and colleagues provided additional insights since they showed that FTO controls the mitotic clonal expansion by interacting with Janus Kinase 2 (JAK2)-Signal Transducer And Activator Of Transcription 3 (STAT3)-C/EBPβ pathway. An increase in m6a, for example caused by FTO depletion, on JAK2 mRNA increases YTH N6-methyladenosine RNA Binding Protein 2 (YTHDF2)-mediated mRNA decay. This leads to reduced JAK2 protein levels with a consequent reduction in STAT3 phosphorylation and C/EBPβ expression, factors that together control the mitotic clonal expansion initiation [61].

Other factors control m6a levels and consequently influence differentiation. For example, the Zinc Finger Protein 217 (ZFP217) affects differentiation due to its ability to interact with Methyltransferase 3, N6-Adenosine-Methyltransferase Complex Catalytic Subunit (METTL3), a key protein responsible for the transfer of methyl groups on mRNA. Loss of ZFP217 inhibits adipogenesis and lipid accumulation and influences the cell cycle by decreasing cyclin D1 (CCND1) levels, ultimately arresting the mitotic clonal expansion. This study observed that ZFP217 interacts with METTL3 and consequently regulates CCND1 expression in an m6a-dependent manner [62].

The observations reported above demonstrate that m6a modification plays a role in adipogenesis and may be a target for further exploitation in future approaches to reduce obesity.

### Cytoskeleton rearrangement at the beginning of adipogenesis

The cytoskeleton forms a complex network composed of different filaments and regulates the cells’ shape and mechanical properties [63]. During adipogenesis, the adipocyte precursors undergo extensive morphological changes through a reorganization of the cytoskeleton, which is associated with a cascade of events leading to a cell shape and structure adequate to accommodate lipid droplets. The transition from fibroblast-like to spherical shape, characteristic of mature adipocytes, is related to a decrease in focal adhesions that stimulates differentiation toward adipocytes [64, 65]. In this process, the state of polymerization/depolymerization of actin has a critical role. Actin adopts a dynamic structure and exists as a globular monomer (G-actin) and a filamentous polymer (F-actin) composed of a linear chain of G-actin subunits. Its dynamism is regulated by numerous signaling pathways and actin-binding proteins (ABPs) [66] and is essential in the remodeling of the cytoskeleton [67]. The intracellular effectors involved in actin dynamics include members of the Rho family of small GTPases (Ras Homolog Family Member A, RhoA; Rac Family Small GTPase 1, Rac1; Cell Division Cycle 42, Cdc42). These factors control actin structures and microtubules and regulate cell shape, polarity, movement, differentiation, and many other fundamental cell processes [68]. The overexpression of RhoA before induction of differentiation results in decreased expression of key adipogenic genes and impairs lipid accumulation, indicating that the regulation of this protein is essential for the proper mature adipocyte formation [69]. Indeed, Rho/Rho Associated Coiled-Coil Containing Protein Kinase 1 (ROCK1) activity is negatively associated with adipocyte differentiation, and the inhibition of this pathway promotes adipogenesis in vitro [70]. Furthermore, a study conducted by Chen and colleagues [71] reported increased amount of G-actin and changes of actin depolymerization factors during differentiation to adipocytes. Their results showed higher levels of unphosphorylated Cofilin 1 (CFL1) and lower protein levels of RhoA and LIM Domain Kinase 1 (LIMK1), which mediates CFL1 phosphorylation, leading to more depolymerized actin and more significant adipocyte differentiation [71].

More in-depth, proliferating mesenchymal stem cells (MSCs) show several F-actin stress fibers throughout the cell body. Upon addition of the adipogenic cocktail, F-actin moves toward the cell’s periphery, which begins to change its shape to a spherical appearance [72]. Factors that can regulate this process also have an impact on adipocyte differentiation. For example, it has been recently reported that the Zinc Finger CCCH-Type Containing 10 (Zc3h10) protein controls the expression of genes involved in cytoskeleton reorganization as early as 36 h after adipogenic program induction. In Zc3h10-depleted preadipocytes, F-actin is less organized, and F-actin microfilaments area is decreased compared to the control group. An opposite situation appears when Zc3h10 is overexpressed. Zc3h10 overexpression enhances F-actin reorganization toward the cell periphery and stimulates lipid droplets formation [72]. In addition, it has been observed that actin organization also plays a key role in determining a discrete subpopulation of functional mitochondria, a key step towards mature adipocyte formation [72].

Another example of how cytoskeleton rearrangements affect the early phase of adipogenesis is provided by the protein Dihydropyrimidinase Like 2 (DPYSL2, also known as CRMP2), a tubulin-binding protein known to provide microtubule assembly [73]. This protein co-localizes with actin filaments, thus confirming its role in cytoskeleton remodeling. During the adipogenic process, the expression of the full isoform (f-CRMP2) remains constant while that of the small isoform (s-CRMP2), which is higher at the day of differentiation induction, then decreases. s-CRMP2 does not exhibit tubulin-binding activity, and the correct balance between f-CRMP2 and s-CRMP2 controls proliferation and the mitotic clonal expansion. Overexpression of CRMP2 causes a decrease in lipid accumulation and the number of cells indicating that this protein also plays a role in the mitotic clonal expansion. Moreover, overexpression of this protein may cause an alteration in microtubule stability, affecting cytoskeleton dynamics and causing a block in the morphological change of the cells that become unable to reach the correct spherical shape typical of mature adipocytes. On the contrary, CRMP2 knockdown causes a decrease in actin polymerization in the cortical portion of the cell, favoring lipid accumulation [73]. The list of different factors involved in cytoskeleton rearrangement during adipogenesis is recapitulated in Table 4.

**Table 4:** Summary of pro- and anti-adipogenic factors that control the activity of cytoskeleton

Protein	Description	References
RhoA	It is a GTPase protein belonging to the Rho family of GTPases involved in the regulation of actin cytoskeleton polymerization. Its overexpression before adipogenesis induction results in decreased expression of key adipogenic genes and reduced lipid accumulation	[69]
ROCK1	It is a major downstream effecter of RhoA and is a regulator of the actomyosin cytoskeleton. It is negatively associated with adipocyte differentiation, and the inhibition of this pathway promotes adipogenesis in vitro	[70]
Zc3h10	A transcription factor involved in the control of energy metabolism and mitochondrial function; its downregulation impairs F-actin cytoskeleton reorganization during the early adipogenesis	[72]
DPYSL2/CRMP2	It is known to be present as a full and a small isoform. The small isoform is higher at the day of differentiation induction and then decreases. It does not exhibit tubulin-binding activity, and the correct balance between f-CRMP2 and s-CRMP2 has been shown to control the proliferation and mitotic clonal expansion	[73]

### Other factors and processes involved in the regulation of the early phase of adipogenesis

RNA metabolism influences several cellular processes, including adipogenesis. An example is ELAV Like RNA Binding Protein 1 (Elavl1, also known as Hur) capable of controlling mRNA stability and translation, and whose expression decreases during differentiation [74]. Hur depletion before the onset of the adipogenic program increases the expression of adipogenic marker genes and lipid accumulation, while its overexpression causes an opposite effect, indicating that this protein has an impact on adipocyte differentiation [74]. A previous study showed that Hur acts at the beginning of differentiation by inhibiting the expression of C/EBP and thus blocking the adipogenic process [75]. Hur's effect has also been confirmed in vivo, where it has been shown that Hur-KO mice display increased fat mass, glucose intolerance, and insulin resistance. Mechanistically, Hur accomplishes its function by acting on the mRNA metabolism of its target genes. Indeed, Hur stabilizes Insulin Induced Gene 1 (Insig1) mRNA, which has a negative effect on adipogenesis [74].

Also, RNA decay is important in adipogenesis, and it occurs in processing bodies (PBs), which are membrane-free organelles responsible for RNA metabolism. The two essential components of these PBs are the proteins DEAD-Box Helicase 6 (Ddx6) and Eukaryotic Translation Initiation Factor 4E Nuclear Import Factor 1 (EIF4ENIF1, also known as 4E-T). When Ddx6 is silenced, 3T3-L1 cells fail to differentiate, and both lipid droplets and PBs are not formed. Thus, the authors suggested that mRNA degradation before differentiation induction is essential for proper adipogenesis since it is necessary to remove the transcriptome of the parental phenotype and generate a new transcriptome allowing the transformation of the phenotype [76].

**Fig. 1:**
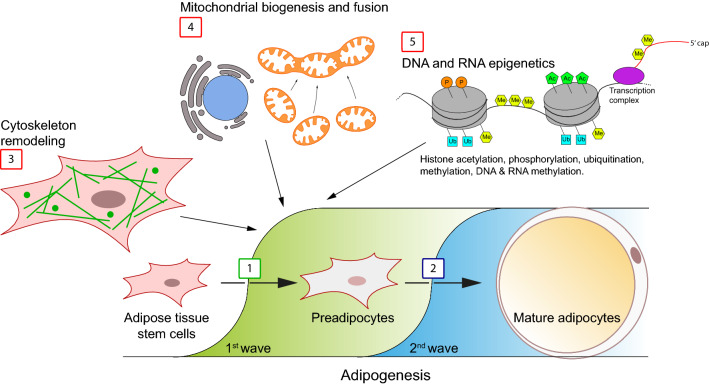
Schematic representation of adipogenesis and of main mechanisms involved in adipose tissue stem cell commitment. In the first phase of adipogenesis, adipose tissue stem cells undergo adipogenic commitment in the presence of specific chemical and physical cues (step 1, green area), while in the second phase white preadipocytes build up lipids as energy storage and express adipokines (step 2, blue area). These steps are mainly controlled by several transcription factors that are finely coordinated in a time-dependent fashion [39, 117–119]. Notably, adipose tissue stem cell transition to preadipocytes is not associated to significant morphological changes, rather to biochemical, genetic, and metabolic rearrangements; these include F-actin fibers breakdown and F-actin rearrangement to preadipocyte periphery (step 3), mitochondrial biogenesis and fusion associated to increased metabolic performance (step 4), and epigenetic events occurring on both DNA, histones and RNA (step 5). Among the main epigenetic modification, histone phosphorylation, acetylation, methylation and ubiquitination together with DNA and RNA methylation have been demonstrated as important regulatory steps of adipogenesis [72, 120, 121]

Another process involved in the differentiation of white adipocytes is autophagy, a cellular mechanism of selective removal of damaged cytoplasmic components. A study conducted by Skop and colleagues [77] showed that this process is important in the initial phase of differentiation while its importance decreases at the late stage of adipogenesis. They hypothesized that autophagy allows the elimination of mitochondria from preadipocytes and causes the subsequent remodeling necessary for proper adipogenesis [77]. Furthermore, recently, the role of Rac Family Small GTPase 3 (RAC3) on adipocyte differentiation and autophagy has been studied. The downregulation of RAC3 expression causes an increase in autophagy and the arrest of the cell cycle, thus accelerating the formation of lipid droplets and the process of adipogenesis [78]. These findings are depicted in Fig. 1.

## Different models to study the early phase of adipogenesis in vitro and in vivo

As already defined, adipogenesis is the process that allows the formation of mature adipocytes from precursor cells such as mesenchymal stem cells. To date, there are several in vitro models to investigate and dissect the molecular mechanisms underlying the adipogenic process. Upon normal culturing conditions, these cells present a fibroblast-like morphology. The current protocols for adipocyte differentiation involve growing cells to confluence and then exposing them to adipogenic stimuli that impose a change in phenotype resulting in achieving spherical shape, lipid accumulation, and acquisition of mature adipocyte characteristics.

The most frequently used in vitro model to study adipogenesis remains the 3T3-L1 cells, immortalized murine cells derived from Swiss 3T3 mouse embryos. They can differentiate from fibroblasts to adipocytes using simple protocols available in the literature [79]. These cells are already committed to the adipocyte lineage and spontaneously differentiate over several weeks into adipocytes when cultivated in the presence of 10% fetal calf serum. However, this process can be accelerated using the so-called “differentiation cocktail”, containing the synthetic glucocorticoid dexamethasone, the phosphodiesterase inhibitor Isobutylmethylxanthine (IBMX), and high insulin concentration [80].

Another in vitro system utilized to study adipogenesis is the 3T3-F442A cell line, which represents a model with a more advanced commitment than 3T3-L1, and therefore does not require the addition of glucocorticoids to the differentiation cocktail [80].

Among the immortalized murine cells, the multipotent stem cell line, C3H10T1/2, is also used. They derive from C3H mouse embryos, and after reaching confluence, do not spontaneously differentiate to adipocytes; to trigger differentiation, it is necessary to use the bone morphogenic protein 4 (BMP4) or the adipogenic cocktail containing glucocorticoids, IBMX, insulin, and PPARγ agonist [81].

Adipocyte precursors can also be isolated from WAT of various species (i.e., human, mouse). They are mesenchymal stem cells isolated from fresh adipose tissue of mice upon collagenase digestion and centrifugation steps. The pellet containing the stromal vascular fraction (SVF) is recovered, and then adipocytes precursors can be cultivated and differentiated in vitro into mature adipocytes [82]. The use of primary adipose tissue stem cells represents a more physiological model to study the process of adipogenesis. However, their isolation requires a conspicuous amount of tissue to have a good yield of preadipocytes. Furthermore, they have a short and limited life span in culture, high variability in results and the age of animals can negatively affect the terminal differentiation. Recently, a different protocol has been developed and involves the isolation of preadipocytes from the WAT or the BAT of newborn pups. Adipose tissue of newborn pups is rich in proliferating adipocyte progenitors compared to adult mice and, in addition, displays greater differentiation potential [83]. Additional cell lines are reported in the literature for the study of adipogenesis in vitro [84, 85].

The models described above are also defined as two-dimensional (2D) models and are systems used to study adipose tissue biology and the beginning of differentiation. However, they cannot replicate the complexity of the in vivo process and lead to the formation of incompletely mature adipocytes that accumulate lipids in small multi-locular lipid droplets, not conforming to what occurs in vivo, namely the formation of a single large lipid droplet.

An alternative method to study adipogenesis in vitro is the generation of a 3D cell culture model of adipocyte precursors. For example, Klingelhutz and colleagues have developed a 3D culture method in which adipocyte precursors isolated from tissue or immortalized cells can self-organize into spheroids from hanging droplets and subsequently differentiate using adipogenic stimuli. In these spheroids, adipocyte precursors differentiate to mature adipocytes more effectively, as assessed by deposition of large lipid droplets, produce more adipokines than 2D culture and maintain white and brown differential gene expression [86]. Another research group has applied a different strategy to produce adipose spheroids and cultivate human adipose-derived stem cells; they use a coating of elastin-like polypeptide-polyethyleneimine copolymer, which allows the generation of mature adipocytes after differentiation induction in a 3D manner [87]. These are just a few examples of the different methods used to generate a 3D culture system, but other technologies have been developed in this field. The advantage of this approach is that, independently from the technology applied, in a 3D model there is an increase in the levels of differentiation and a greater complexity resembling that observed in vivo. However, these techniques are more challenging to develop and more expensive than 2D cultures.

Recently, single-cell RNAseq technology has also been used to study adipogenesis. As a result, it has been discovered that at least two distinct classes of subcutaneous white adipocytes exist. These differences in gene expression are reflected in their differentiation and mainly affect pathways involved in the cell cycle, protein synthesis, and growth. Therefore, using this innovative technique is very useful to investigate the different adipogenic processes in different adipose depots and the variability of single cells within the tissue [88].

Finally, the in vivo study of adipogenesis is much more complex and expensive. The Wagyu cattle can be used due to their exceptional intramuscular fat deposition [89]; in addition, Wang and Scherer [90] developed the AdipoChaser mouse which allows tracking the adipogenic process in vivo using a specific transgenic mouse.

## Adipose tissue hypertrophy and hyperplasia in obesity

Obesity has medical, social, and economic impact that predisposes to a number of disabilities, comorbidities, and deaths [91]. Obese subjects are affected by a low-grade and chronic inflammation in both BAT and WAT [92, 93]. Central obesity, due to excessive accumulation of dysfunctional visceral WAT (vWAT), correlates with the onset of type 2 diabetes (T2D) and cardiovascular and cerebrovascular events [92]. In this context, the role of adipose tissue plasticity of different fat depots in the onset of adverse metabolic phenotypes has been recently demonstrated [94–96]. The dynamics of adipose tissue mainly relies on two processes, namely adipocyte hypertrophy and hyperplasia, where the former refers to the enlargement of previously existing fat cells (i.e., hypertrophic adipose tissue), while the latter is the consequence of the excessive generation of new adipocytes through adipogenesis (hyperplastic adipose tissue) [97]. Hypertrophic and hyperplastic fat depots have been observed in sWAT and vWAT of normoglycemic, prediabetic and T2DM patients with obesity compared to lean subjects [98]. In addition, the authors found that sWAT preadipocytes collected from obese subjects have decreased adipogenic potential compared to those obtained from lean controls, while no difference was observed in the adipogenic potential of vWAT precursors [98]. This finding suggests that adipogenesis in sWAT, but not in vWAT, represents a protective mechanism to cope with excessive food intake. In line with this observation, other studies showed that adipocyte number in sWAT is positively correlated with metabolic phenotypes, i.e., insulin sensitivity and HDL-cholesterol, and negatively associated with blood insulin and triglyceride levels, independently of fat mass [96, 99, 100]. On the other hand, vWAT hypertrophy and hyperplasia have been linked to obesity and metabolic dysfunction [98, 101]. Other studies showed that metabolically healthy obese subjects have reduced vWAT adipocyte size, while increased adipocyte size in omental adipose tissue is associated to adverse metabolic phenotype, and possibly progression from hepatic steatosis to fibrosis [102, 103]. On the contrary, an independent investigation indicated that excessive accumulation of dysfunctional mesenteric, but not omental adipose tissue, plays a role in the onset of diabetes and hepatic steatosis in obese subjects [104]. Together, these observations suggest that sWAT, but not vWAT, adipogenesis may represent a protective process against obesity and the onset of unfavorable metabolic phenotypes. Further investigations need to assess if the over and under representation of specific adipogenic cell lineages within different or even the same fat depot may represent a risk factor for the susceptibility to obesity and other metabolic diseases. Table 5 summarizes the main findings about adipose tissue heterogeneity and association to metabolic phenotypes.

**Table 5:** Summary of adipose tissue heterogeneity and association to metabolic phenotypes

Adipose tissue	Description	Reference
Subcutaneous and visceral adipose tissues	Hypertrophy and hyperplasia in normoglycemic, prediabetic and T2DM patients with obesity Largest increase in adipocyte size and decrease in adipose tissue stem cells number and adipogenic potential in T2DM and in prediabetes sWAT preadipocytes from obese subjects show decreased adipogenic potential compared to lean controls No difference in the adipogenic potential of vWAT precursors in obese subjects compared to lean controls	[98, 101]
Subcutaneous adipose tissue	Adipocyte number positively associates with insulin sensitivity and HDL-cholesterol, and negatively associate with blood insulin and triglyceride levels	[96, 99, 100]
Omental adipose tissue	Metabolically healthy obese subjects have reduced vWAT adipocyte size Adverse metabolic phenotype, progression from hepatic steatosis to fibrosis correlate with increased adipocyte hypertrophy	[102, 103]
Mesenteric adipose tissue	Mesenteric, but not omental nor subcutaneous adipose tissue dysfunction associates with the onset of diabetes and hepatic steatosis in obese subjects	[104]

## Perspectives and concluding remarks

The growing understanding of the molecular mechanisms underlying adipogenesis is shedding new light on our awareness of the role of adipose tissue in pathophysiology. New technologies and integrated omics approaches are instrumental to reveal how RNA transcription, protein translation, proteomic and metabolomic patterns orchestrate adipogenesis and contribute to adipose tissue phenotypes. However, several gaps need to be further explored at both preclinical and clinical level:

- The mechanisms underlying the regulation of adipose tissue stem cell stemness and the molecular requirements for commitment to preadipocyte. In this context, the identification of novel cell surface markers is crucial for the isolation and characterization of adipose tissue stem cells to better understand their contribution to the development of fat subtype(s).
- It has been shown that perilipid mitochondria are different from cytoplasmic mitochondria both morphologically and functionally [105], and another work demonstrated how mitochondrial morphology changes during adipogenesis [72]. There is a need to investigate how mitochondria and other intracellular organelles, such as cytoskeleton, the endoplasmic reticulum, lysosomes, autophagosome, or nucleus, contribute to coordinate different phases of adipogenesis.
- Adipogenesis is a time-dependent process. For this reason, it is necessary to understand the processes that take place during the different stages of adipocyte differentiation. Characterizing and then stratifying these molecular mechanisms would allow to prioritize new possible targets to be further exploited for innovative pharmacological treatment of adipose tissue-related diseases.
- The balance between adipocyte subtypes in adipose tissues or even within the same fat depot, together with the proper interaction of adipocytes with other cell populations, are crucial requirements to guarantee the optimal tissue homeostasis [106, 107]. The constant progression of single-cell-based analyses should be further exploited to enrich our knowledge on cell population heterogeneity and cell population interactions in several contexts, such as adipose tissue development, hyperplasia, hypertrophy, fibrosis and other adipogenesis-related pathologies.
- Adipose tissues exhibit distinctive characteristics based on their body distribution. The differences between BAT and WAT have been extensively described in the literature; however, other fat subtypes have been identified with distinctive characteristics [106, 108–110]. Characterizing the role of known and eventually identifying new adipose tissue subtypes is mandatory to understand the etiology and progression of obesity-related diseases and ultimately implement personalized medicine programs.
- Currently, available medications for obesity include orlistat, phentermine/topiramate, lorcaserin, naltrexone/bupropion, and liraglutide [111, 112]. Despite the efficacy of these molecules on obesity is well established, less is known about their role on adipogenesis. For instance, glucagon-like peptide-1 analogs and sodium-glucose transport protein 2 inhibitors (SGLT2i) have been tested both in vitro and in vivo as adipogenesis modulators, but their exact action in this context is still unclear [113–116]. Improving our knowledge of the existing therapeutic solutions for obesity, especially regarding their impact on adipogenesis and mechanisms such as hyperplasia and hypertrophy, is a mandatory task to pave the way towards innovative therapeutical solutions.

## Data Availability

Not applicable.
